# The role of the inferior frontal gyrus in vicarious social touch: A transcranial direct current stimulation (tDCS) study

**DOI:** 10.1016/j.dcn.2018.04.010

**Published:** 2018-04-30

**Authors:** Leehe Peled-Avron, Laura Glasner, Hila Z. Gvirts, Simone G. Shamay-Tsoory

**Affiliations:** a Department of Psychology, University of Haifa, Haifa, Israel; b Department of Behavioral Sciences and Psychology, Ariel University, Ariel, Israel

**Keywords:** Social touch, tDCS, Inferior frontal gyrus, Empathy, Interpersonal touch, Empathic concern

## Abstract

The neural mechanisms facilitating the experience of vicarious social touch are largely unknown. The right inferior frontal gyrus (rIFG) has been suggested as part of a simulation observation-execution neural network that plays a key role in the perception of tactile stimuli. Considering that vicarious social touch involves vicarious sharing of emotions, we hypothesized that emotional empathy, i.e., the ability to feel what another individual is feeling, modulates the neural responses to vicarious touch. To examine the role of the rIFG in vicarious touch and its modulation by levels of emotional empathy, we used anodal transcranial direct current stimulation (tDCS) on forty participants who observed photos depicting social touch, nonsocial touch or no touch during tDCS or sham stimulation. The results show that while participants with high levels of emotional empathy exhibited no change in ratings of vicarious social touch, participants with low levels of emotional empathy rate human touch as more emotional following anodal stimulation of the rIFG than following sham stimulation. These findings indicate that emotional responses to vicarious social touch are associated with rIFG activity and are modulated by levels of emotional empathy. This result has major therapeutic potential for individuals with low empathic abilities, such as those with ASD.

## 1 Introduction

Social touch encompasses a large variety of behaviors that involve physical contact between humans, ranging from positive and affective gestures of touch through neutral, accidental or functional touch to negative touch that includes violence (Gallace and Spence, 2010). In this study, we focused on positive affiliative and affective touch between humans. Our first lessons in loving come from parental touch, which we receive as infants (Harlow et al., 1965; Harlow and Zimmermann, 1959). Throughout life, gestures of positive social touch such as hand-holding, hugs or gentle caresses serve as a powerful means of eliciting and modulating our feelings and emotions (Hertenstein et al., 2009, 2006; Kirsch et al., 2017). Furthermore, touch is used to enhance the meaning of other forms of verbal and non-verbal communication (Gallace and Spence, 2010). A recent large cross-cultural study showed that human social touch reflects an essential mechanism supporting the maintenance of social bonds (Suvilehto et al., 2015).

Schirmer et al. (2015) showed that the mere observation of social touch is associated with positive emotions and that characters portrayed in photos seem more positive and likeable when they touch each other than when they do not. These intriguing findings indicate that third-party observers of touch simulate the feeling of being touched and are thus able to understand and identify with the social experience of the recipient. In line with this view, Keysers et al. (2004) proposed the existence of a neural simulation system for observed touch. Using functional magnetic resonance imaging (fMRI), these researchers showed that observation of another person's leg being touched by a stick resulted in neural activity in the secondary somatosensory cortex (SII). Another fMRI study by Blakemore et al. (2005) revealed that observation of touch elicited activity in both primary (SI) and secondary (SII) somatosensory cortices. In particular, the activation was somatotopically organized and different regions were activated when the observed touch was to the person's neck or face (Blakemore et al., 2005). Pihko et al. (2010) also reported SII activation while participants observed the experimenter touching her own hand during the experiment. Taken together, these results suggest that social touch engenders mental somatosensory simulation in those who observe it.

A possible mechanism that may facilitate this experience of vicarious social touch in a third-party observer is empathy. Empathy is defined as the way in which an individual reacts to the observed experiences of another (Davis, 1983). Researchers have increasingly acknowledged the existence of two main systems of empathy: an emotional empathy system that supports our ability to resonate with other people's mental and physical states, and a cognitive perspective-taking system that involves adopting the other's point of view (de Waal, 2008; Shamay-Tsoory, 2011; Shamay-Tsoory et al., 2009). Vicarious social touch, which involves shared feelings of touch, is likely to be modulated by the emotional empathy system, since sharing the other's embodied and emotional state unconsciously activates emotion-generation mechanisms (Adolphs, 2002, 2003; Adolphs et al., 2000). Indeed, several studies have shown that levels of neural activity in response to vicarious touch are correlated with levels of empathy (Cheng et al., 2008; Gazzola et al., 2006; Schaefer et al., 2012; Voos et al., 2013). Therefore, it is likely that vicarious social touch and emotional empathy share some neural mechanisms.

A core region in the emotional empathy network is the Inferior Frontal Gyrus (IFG) (Seitz et al., 2008; Iacoboni, 2009; Farrow et al., 2001; Schulte-Rüther et al., 2007; Jabbi and Keysers, 2008). The IFG is marked by a certain hemispheric asymmetry regarding simulation mechanisms. The left IFG is widely known to possess motor simulation characteristics and was found to contain a motor representation of hand, arm and mouth movements (Binkofski et al., 1999; Ehrsson et al., 2000; Gerardin et al., 2000; Iacoboni et al., 1999; Krams et al., 1998; Buccino et al., 2001). The right IFG (rIFG) was found to play a major role in vicarious gustatory emotions such as hunger and disgust (Jabbi et al., 2007). Moreover, the rIFG was found to be activated during negative experiences occurring to someone else but not to oneself, further accentuating its involvement in emotional empathy (Perry et al., 2012). The cortical thickness of the rIFG was positively correlated with empathic abilities (Massey et al., 2017), and individuals with schizophrenia who have especially low levels of emotional empathy exhibited reduced cortical thickness, particularly in the rIFG. In line with these findings, impaired function of the rIFG has been found in several developmental disorders characterized by deficient empathetic capacities (e.g., autism spectrum disorders) (Greene et al., 2011; Grezes et al., 2009).

Interestingly, the rIFG was found to relate to tactile processing in general. A study that examined subjective, behavioral and neural processing during tactile stimulation using a soft brush stroke found that the rIFG was activated in both adolescents and young adults (May et al., 2014). Furthermore, the rIFG has been implicated both in tactile object recognition and in tactile object localization processes and as such is considered an integral part of a neural network responsible for tactile processing (Reed et al., 2005). In a case study of a 36-year-old schizophrenic patient, tactile somatic hallucinations activated the rIFG along with the precuneus area and the posterior cingulate gyrus (Shergill et al., 2001).

The rIFG also appears to be implicated in various emotional empathic functions on the one hand, and in tactile processing on the other hand. Nevertheless, no study to date has examined its involvement in vicarious social touch *and* its modulation by levels of emotional empathy. We therefore sought to examine whether deliberately manipulating rIFG excitability would directly augment levels of vicarious touch/tactile empathy in individuals who have general difficulties in emotional empathy.

In order to increase levels of rIFG excitability, we used non-invasive brain stimulation by means of anodal transcranial direct current stimulation (tDCS). tDCS alters neuronal membrane potentials, thereby modulating the levels of excitability of a targeted region (Bindman et al., 1962; Zheng et al., 2011). Anodal tDCS stimulation has been reported to increase cortical excitability (Nitsche et al., 2003; Nitsche and Paulus, 2001), while cathodal stimulation decreases cortical excitability. However, accumulated data reveals that while anodal stimulation reliably increases cortical excitability, cathodal stimulation was often found not to induce any consistent changes in cortical excitability (Dyke et al., 2016). Hence, in this study we focused on anodal stimulation.

We hypothesized that individuals with low emotional empathy levels would show an increase in their emotional ratings of vicarious touch following anodal stimulation of the rIFG. For this purpose, we screened forty participants with either high or low levels of emotional empathy and asked them to rate their level of emotional identification with social touch during anodal stimulation of their rIFG.

## 2 Methods

### 2.1 Participants

Forty (18 males) participants took part in the study (mean age: 25.16, s.d: 3.72, range: 20–39; mean years of education: 14.43, s.d: 1.90, range: 12–21). Each participant received either course credit or payment for participating in the experiment. One participant was left-handed and all participants met the inclusion criteria according to brain stimulation protocols (Bikson et al., 2009; Nitsche et al., 2003). All participants had normal or corrected-to-normal visual acuity and normal hearing. Participants were naïve to the experimental hypothesis and were unaware of the type of stimulation they received in each session. They gave written informed consent prior to inclusion in the study. The study was approved by the University of Haifa Ethics Committee. Two participants were excluded from the data analysis since they did not complete the IRI questionnaire properly, and three participants were excluded due to ratings that exceeded the average rating by more than three standard deviations. Hence, the reported results are based on 35 participants (16 males). Prior to the experiment, each participant completed the interpersonal reactivity index (IRI) questionnaire in order to assess level of emotional empathy as reflected in the emotional concern (EC) subscale of the questionnaire. Based on whether their average EC score^1^ was above or below the group’s median score of 3.85 (s.d = 0.659), the participants were further classified into a low emotional empathy group (EC < 3.85; N = 17 participants) or a high emotional empathy group (EC ≥ 3.85; N = 18 participants). The mean EC scores in the low and high emotional empathy groups were 3.09 ± 0.45 and 4.35 ± 0.37, respectively.

### 2.2 Stimuli, task and design

**Fig. 1:**
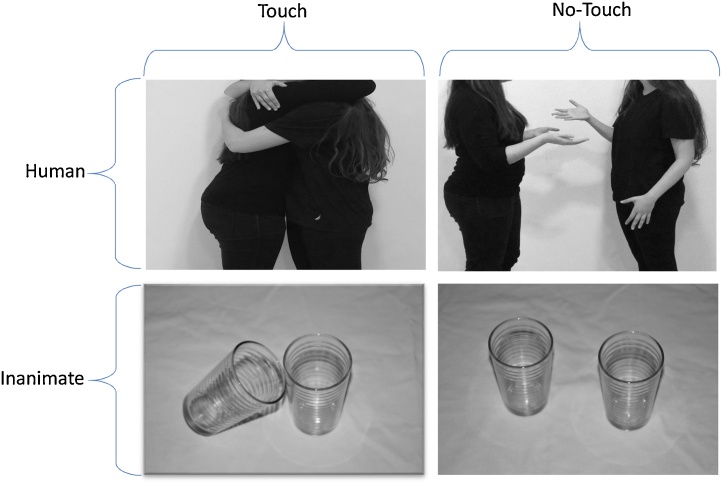
Photos illustrating each of the four conditions explored in the study. Note that both inanimate objects and humans were photographed against a white background. All humans wore black clothing and were photographed from the shoulders down in order to avoid the confounding effects of facial expressions.

A variation of this task has been used in previous studies (Peled-Avron et al., 2016a,b). The current study used a randomized, single-blind, sham-controlled, within-subject design. Participants sat approximately 60 cm from a 21″ flat screen monitor and were asked to complete a computerized task (E-Prime 2.2 Psychological Software Tools was used for stimulus presentation and experimental control). The participants were shown monochromatic images, all sized 6″ × 4″ (∼15 cm × 10 cm) at landscape orientation with fixed luminance in order to control for possible low-level visual differences between the stimulus categories (Johannes et al., 1995). The participants were shown 80 photos of four different conditions: human touch, human non-touch, inanimate touch and inanimate non-touch. The human touch condition contained photos depicting various types of social touch, including a hug, a handshake or friendly hand-holding. The inanimate touch condition included photos depicting two everyday objects (without any commercial logos) touching each other and positioned in various ways. The other two conditions presented the same humans or objects in proximity to one another but not touching (Fig. 1).

### 2.3 Procedure

Prior to participation in the experiment, each participant completed the Hebrew version of the IRI questionnaire (Davis, 1983). This version has been translated into Hebrew and validated (Even, 1993). The IRI is a 28-item self-report measure consisting of four 7-item subscales, each tapping a different aspect of the global concept of empathy, broadly defined as a measure of reactivity to others. As our a priori hypothesis pertained solely to the empathic concern scale, we focused on this subscale of the questionnaire, which assesses feelings of sympathy and concern for unfortunate others.

Each participant underwent two stimulation sessions separated by a one-week interval. The order of stimulations was counterbalanced across participants. Each session included one type of stimulation: either sham or anodal (excitatory) stimulation. The study was a single-blind experiment. The participants were not aware of the type of stimulation they received, while the experimenter was fully informed (please see Cattaneo et al., 2011; Pisoni et al., 2012 for similar procedures). The task commenced three minutes after the onset of stimulation, as studies have shown that cortical excitability changes due to tDCS are observed after three minutes of stimulation (Nitsche and Paulus, 2001; Nitsche and Bikson, 2017)

The stimuli were presented in four blocks of 20 trials each, for a total of 80 trials. Blocks were randomized across stimulation conditions and participants. A block design was used in order to allow for intermissions in the task. These intermissions were included to allow the participants to rest and state whether they had experienced any discomfort during the electrical stimulation. In addition, three practice trials were carried out during the instructions to ensure that each participant fully understood the method and meaning of the ratings. In each trial, the participants were shown a picture of two inanimate objects or two humans. In each picture, the objects or humans were either touching or not touching but in close proximity to one another. The participants were instructed to rate the emotional level of each photo using a visual analog scale (VAS) that ranged from “not emotional” to “very emotional.” Each trial consisted of a fixation cross shown for 500 ms, followed by a photograph with a VAS below it shown for the duration of the ratings. An inter-trial interval of a blank screen was presented for 400 ms.

### 2.4 tDCS

Stimulation was delivered by a battery-driven constant current stimulator (Magstim, Whitland, Wales, UK) through two saline-soaked sponge electrodes (experimental electrode: 25 cm^2^ 5 × 5, reference electrode: 35 cm^2^ 7 × 5) that were placed on the head and kept in place with textile straps. A constant current of 1.5 mA was applied for 15 min. Participants performed the task online during the stimulation or sham condition. The task lasted approximately 10–12 min, including practice sessions and intermissions. To ensure homogeneity of stimulation length across participants, any participant that finished the task in less than 15 min was asked to remain seated and wait for the experimenter to switch off the device at the end of the 15 min. Localization was established using the 10–20 EEG system. During all stimulation conditions, the experimental electrode was placed on the right IFG, which was determined to be the crossing point between T4-Fz and F8-Cz (Jacobson et al., 2012). The reference electrode was placed above the left frontopolar cortex (Nitsche and Paulus, 2001). In the anodal condition, the anodal electrode served as the experimental electrode and the cathodal as reference. During the sham stimulation, the placement of electrodes remained the same for each participant, but the current was turned off 30 s after the beginning of the stimulation. Participants were debriefed following each session to confirm that they had not been able to distinguish between the sham and the stimulation conditions. In both anodal and sham conditions, the current was turned on and off in a ramp-like fashion for a duration of 7 s (Ambrus et al., 2012; Nitsche et al., 2003) to elicit a transient tingling sensation on the scalp that faded after a few seconds. This procedure ensures the same sensation for both experimental sessions, allowing for successful blinding of participants to the stimulation condition (Gandiga et al., 2006) and preventing participants from abruptly feeling the end of the tDCS protocol.

## 3 Results

A three-way repeated-measures analysis was used, with stimulation (sham, anodal) and task condition (human touch, human non-touch, inanimate touch, inanimate non-touch) as the within-subject factors and emotional empathy (high emotional empathy, low emotional empathy) as the between-subject factor. The degrees of freedom were corrected using Greenhouse–Geisser epsilon values and Bonferroni when necessary. Effect sizes were calculated for all group comparisons in order to determine the magnitude of the group differences.

A main effect of task condition [F (3,99) = 86.557, p < 0.001, p2 = 0.724] was found, with higher emotionality ratings in the human touch condition than in the human non-touch, inanimate touch and inanimate non-touch conditions.

**Fig. 2:**
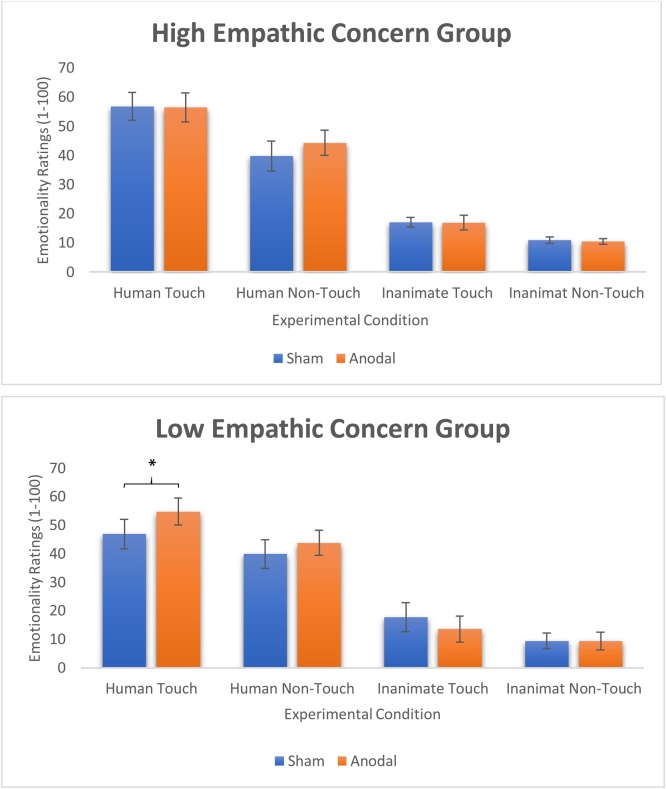
The differential effect of stimulation on the EC groups (top graph shows the high EC group and bottom graph shows the low EC group) for emotional ratings of the photos in all four conditions. Error bars represent the standard error of the mean. * p = 0.048.

Notably, there was a significant third-order interaction between stimulation, condition and emotional empathy scores [F (3,99) = 4.395, p = 0.011, p2 = 0.118]. No other main effects or interactions were found. Follow-up paired t-tests showed a significant difference between the emotionality ratings following sham versus anodal stimulation [t(16) = 2.103, p = 0.048, d = 0.40] for the low emotional empathy group, such that anodal stimulation increased the emotionality ratings in the human touch condition (see Fig. 2). For the high empathy group, however, no such difference was found between the different stimulation conditions [t(17) = 0.495, p = 0.627, n.s., Bonferroni-corrected for multiple comparisons]. Follow-up independent t-tests also revealed that following sham stimulation, the human touch emotionality ratings of the high empathy group (M = 56.83, S.D = 4.73) were higher than those of the low empathy group (M = 46.91, S.D = 5.18) [t(34) = 2.478, p = 0.032, d = 0.42]. However, following anodal stimulation, the human touch ratings of the high empathy group (M = 56.47, S.D = 5.02) did not significantly differ from those of the low empathy group (M = 54.79, S.D = 4.66) [t(34) = 1.35, p = 0.185, n.s.]. No other significant differences were found in any of the other conditions (all P’s > 0.231, Bonferroni-corrected for multiple comparisons) (Fig. 2).

Three-way repeated-measures analyses were used to examine whether the other three subscales of the IRI questionnaire—personal distress, perspective taking and fantasy skills—affected the dependent variable. In the first analysis, stimulation (sham, anodal) and task condition (human touch, human non-touch, inanimate touch, inanimate non-touch) were the within-subject factors and personal distress (high personal distress, low personal distress) was the between-subject factor. No significant effects were found for personal distress (all F’s < 1.906, all p’s > 0.176). In another three-way repeated-measures analysis that used the same within-subject factors and perspective taking (high perspective taking, low perspective taking) as the between-subject factor, no significant effects were found for perspective taking (all F’s < 2.494, all p’s > 0.123). Finally, in a three-way repeated-measures analysis that used the same within-subject factors and fantasy (high fantasy skills, low fantasy skills) as the between-subject factor, no significant effects were found for fantasy skills (all F’s < 3.705, all p’s > 0.082).

## 4 Discussion

In the current study, we showed that anodal tDCS stimulation targeting the rIFG had a differential impact on emotionality ratings of vicarious touch, depending on the emotional empathy levels of the participants. Participants with low emotional empathy rated interpersonal touch as more emotional following anodal tDCS stimulation to the rIFG, whereas no such increase emerged among participants with high emotional empathy. Ultimately, individual differences in empathic capacity, and especially emotional empathic capacity, may modulate the ability to resonate with the somatic feelings of another and are associated with activity in the rIFG.

Our findings correspond to the literature implicating the rIFG in processing touch stimuli (May et al., 2014; Reed et al., 2005; Shergill et al., 2001). Our unique contribution is that the level of rIFG excitability while processing touch stimuli is modulated by individual traits of emotional empathy. Specifically, the ability to simulate and evaluate levels of emotionality of vicarious touch can be enhanced using anodal stimulation of the rIFG, and this enhancement is dependent upon the individual's level of emotional empathy.

Preston and De Waal (2002) proposed a Perception–Action Model, which describes the process by which we understand and empathize with others. According to this model, perceiving the situation of another, automatically activates the perceiver's representations of that situation, which in turn activates the perceiver's responses. As a result of this “shared” neural representation, the perceiver simulates to some extent the feelings felt by the perceived person; thus, enabling a better understanding of the other's internal state. It has been suggested that the neural basis allowing for these simulation processes is a network of mirror neurons. A growing body of research supports the existence of such a network for motoric actions and it has also been shown to play a role in emotional understanding and responses (Rizzolatti et al., 2001). Moreover, accruing evidence provides support for a tactile mirror system comprised of the somatosensory cortices (SI and SII) and areas of the mirror neural system, including the posterior parietal cortex, insula, superior temporal sulcus and the IFG (Gallese, 2001, 2003; Gallese et al., 2004; Blakemore et al., 2005; Keysers et al., 2010; Gordon et al., 2013; Kaiser et al., 2015; Morrison et al., 2011; Morrison et al., 2010). According to this theory, vicarious viewing of interpersonal social touch activates this system. Indeed, several studies found that manipulating the activity of mirror neuron regions such as the somatosensory cortices led to changes in the processing and evaluation of vicarious touch (Bolognini et al., 2013, 2014, 2011). Our results are in line with these studies and further support the existence of a tactile mirror system. Using tDCS we managed to investigate the encapsulated effect of this region and by manipulating the excitability levels of the rIFG, we showed that this area is directly involved in emotional responses to vicarious touch; thereby, contributing to the characterization of the tactile simulation network. In order to further characterize the tactile simulation network and the specific functional role of the rIFG in this network, future studies should apply stimulation-imaging methods and examine the connectivity of the rIFG with other tactile simulation network regions such as the somato-sensory cortices, the posterior parietal cortex and the insula, following tDCs real or sham stimulation.

Our results are in agreement with those reported by Peled-Avron et al. (2016a,b), who showed that vicarious touch can induce varying levels of pleasant emotions depending on individual levels of empathy. In this study, individuals with higher levels of trait empathy exhibited more emotional responses to vicarious touch than individuals with lower levels of trait empathy. These results were found both on the behavioral level, as reflected in the individual's increase in emotional ratings, and on the neural level, as reflected in the individual's degree of suppression in the frequency of mualpha (8–13 Hz) synchronized brain activity. Suppression in the mualpha frequency is largely related to social skills and empathic abilities (Perry and Bentin, 2009; Perry et al., 2010). Our results also conform to those of Perry et al. (2014), who showed that variance in empathy levels modulates the effects of oxytocin administration on interpersonal distance preference. Specifically, among highly empathic individuals, oxytocin decreased the preferred interpersonal distance to reflect physically closer social interactions, whereas for individuals with low empathic traits, oxytocin increased the preferred interpersonal distance to reflect a more distant and avoidant physical distance in social interactions. Our study demonstrates that external manipulation of the excitability levels of a specific brain region involved in empathy—i.e., the rIFG—can improve emotional functions depending upon the individual's empathic levels.

Our results also correspond to those of Fini et al. (2017) who suggested that different individual levels of empathy further interact with the effects of tDCS targeting the left IFG. Specifically, they demonstrated that anodal stimulation of the left IFG increase interpersonal motor resonance among individuals with low scores on the perspective-taking scale of the IRI survey (Davis, 1983). Comparable to our results, the authors found no such increase among individuals with high perspective-taking scores. They suggested that since the individuals scoring high on perspective-taking showed high interpersonal motor resonance skills to begin with, stimulation could not further improve their performance. In line with this, we found that individuals with low emotional empathy levels exhibited an increase in their emotional ratings of vicarious touch following an anodal stimulation while no change was observed in individuals with high emotional empathy. This recurring differentiation between the high and low emotional empathy groups further substantiates the claim that the rIFG is involved in vicarious touch and that manipulating the activity of this region may aid in raising empathic abilities and improve social functioning in those with disorders characterized by low empathic abilities, such as ASD.

In addition to our analyses of the empathic concern subscale of the IRI questionnaire, we explored the unique contribution of the other IRI subscales to the behavioral responses of tactile empathy. We did not find any effects for the other subscales, namely personal distress, perspective taking and fantasy skills. These results further substantiate and strengthen our preliminary assumption that emotional empathy is the primary aspect of empathy involved in tactile resonance and rIFG activation.

It is noteworthy that we found enhancement in ratings of emotionality only for vicarious touch but not for the inanimate touch or non-touch conditions. Since the IFG has been implicated in the motor observation-execution system (Iacoboni, 2009; Shamay-Tsoory, 2011; Shamay-Tsoory et al., 2009; Dvash and Shamay-Tsoory, 2014), one might assume that the human touch condition, which includes explicit motor actions, would be affected by IFG stimulation. In our study, however, both the human touch condition and the human non-touch condition included explicit motor actions, since both were photographed during movement. Moreover, the non-touch photos contained even more movement than the touch photos since touch is relatively still whereas a conversation usually includes waving and moving the upper body parts to express communication. Therefore, our results do not pertain solely to motor imitation or simulation but also to empathic aspects of simulation. Nevertheless, since the IFG is mainly a sensorimotor circuit and since sensation and action are two sides of a very thin coin in this circuit, direct experimental controls are needed to rule out any covariance between emotion measures and intention/goal inference. We acknowledge this lack of experimental controls in our experiment as a limitation to be addressed in future research on this subject.

Furthermore, the present study paradigm did not include a nonsocial touch control condition between a human and an inanimate object since it was previously shown that the sight of an unintentional, non-meaningful or accidental touch between a human and an object elicits less activation in tactile brain areas than intentional and meaningful touch between humans (Ebisch et al., 2011, 2008). Hence, we decided to omit such conditions in our study in order to focus on the response to meaningful social affective touch and its modulation by individual emotional empathy levels. Future studies would benefit from researching the involvement of the rIFG in aspects of nonsocial touch between a human and an inanimate object.

It is important to note that the sample size of each group (low and high empathy) is small and therefore, larger studies are needed to further substantiate our findings. Nevertheless, a medium effect size was found when subjects with high empathy were compared with subjects with low empathy who received an anodal stimulation to the rIFG, on human touch emotional ratings, thus, indicating that the magnitude of the group differences is considerable and merits further research.

A limitation of this study is that we used only anodal stimulation compared to sham and did not examine the effects of cathodal stimulation. We chose to focus on the effects of anodal stimulation since it has been consistently reported to reliably and significantly increase cortical excitability levels compared to sham (Dyke et al., 2016; Jacobson et al., 2012; Nitsche and Paulus, 2001). Cathodal stimulation, in contrast, has been reported both as *decreasing* cortical excitability (Nitsche et al., 2003; Nitsche and Paulus, 2001) and as *increasing* cortical excitability (Batsikadze et al., 2013; Jacobson et al., 2012). Furthermore, one recent study even found that cathodal stimulation neither decreased nor increased cortical excitability (Dyke et al., 2016). Due to the conflicting results pertaining to the nature of cathodal stimulation, we decided to examine anodal stimulation, which also conformed to our hypothesis. Future research using different methodology (e.g., TMS) is warranted to investigate the effects of inhibitory versus excitatory stimulation of the rIFG on vicarious social touch with regard to empathy levels.

Moreover, it is important to note that in this study, the task commenced three minutes after the onset of stimulation. This choice was based on evidence demonstrating excitability changes of up to 40% in the motor cortex 3–5 min following onset of stimulation (Nitsche and Paulus, 2000), 2001. Nevertheless, the literature includes conflicting reports regarding the time course of tDCS effects. For instance, a magnetic resonance spectroscopy study of GABA did not find excitability changes during 30 min of anodal stimulation. The authors showed that tDCS effects developed during stimulation emerge only 10–15 min after termination of stimulation and persist for 20 min (Bachtiar et al., 2015). The findings of Kuo et al. (2013) are also in line with this notion, showing a delayed peak excitability of the motor cortex at 30 min post-anodal stimulation. In studies incorporating simultaneous application of tDCS and magnetoencephalography, there were no online changes in average power within the visual gamma and alpha frequencies (Hanley et al., 2016; Marshall et al., 2016). Nevertheless, studies focusing on frequency changes found significant effects of anodal stimulation compared to sham, 20–30 min post stimulation (Heinrichs-Graham et al., 2017; Wilson et al., 2017). Studies incorporating simultaneous application of tDCS and fMRI yielded conflicting results. Resting state spontaneous activity showed profound differences during tDCS stimulation and sham (Callan et al., 2016). Similarly, specific online tDCS effects on neural activity revealed a task-dependent change in rIFG activation (Hauser et al., 2016). Several studies found effects after the offset of stimulation in cerebellar regions (D’Mello et al., 2017) and motor cortices compared to sham stimulation (Waters et al., 2017). Lastly, Alekseichuk et al. (2016) found that anodal tDCS over the visual cortex induced an increase in BOLD responses evoked by visual stimuli during stimulation but found no effect after cessation of stimulation. Review of the current tDCS-fMRI results suggests that there is great variation in the manner in which tDCS techniques are employed and that its effects are largely task dependent.

In conclusion, in this study we show that anodal stimulation of the rIFG increases ratings of emotionality for observed vicarious touch, depending on levels of emotional empathy. Our study contributes to the research field of social and emotional touch both from a theoretical and a clinical point of view. Our study demonstrates that the rIFG is directly involved in simulation mechanisms of the somatosensory perception system and as such contributes to the mapping of the tactile neural mirror network to include the rIFG. Future research should examine brain connectivity to characterize the relationship between the rIFG and other tactile mirroring areas, such as the somatosensory cortices, during vicarious touch. From a clinical perspective, we show here that *the level* of rIFG excitability is modulated by individual traits; thus, demonstrating that the neural mirror network is inhomogeneous across individuals and even external manipulations such as tDCS, have varying effects depending on the individual receiving the treatment. Future studies should investigate the effects of an anodal stimulation of the rIFG on emotional ratings of vicarious touch in clinical populations characterized by impaired emotional empathy, such as autism spectrum disorders (Dziobek et al., 2008) and schizophrenia (Lee et al., 2004).

With regard to the social domain in which affective states can be and usually are evoked by touch, people can project themselves into the tactile situation faced by another person through the simulation mechanism supported by the rIFG. By the same token, the cortical excitability of the rIFG and innate levels of emotional empathy may account for the quantity and quality of vicarious experiences in our social environment.

## Conflict of Interest

None.

## Footnotes

1 EC distribution data: Mean = 3.710; SEM = 0.124; Mode = 3.43; Range = 3.29; Minimum = 1.71; Maximum = 5.00.
